# Dynamic Scoring: Probabilistic Model Selection Based on Utility Maximization

**DOI:** 10.3390/e21010036

**Published:** 2019-01-08

**Authors:** Jan Vecer

**Affiliations:** 1 Department of Probability and Mathematical Statistics, Charles University, Sokolovska 83, 18675 Praha, Czech Republic; vecer@karlin.mff.cuni.cz; Tel.: +420-221-913-419; 2 Vysoka Skola Aplikovaneho Prava, Chomutovicka 1443, 14900 Praha, Czech Republic

**Keywords:** model selection, utility maximization, explicit supply and demand functions, equilibrium, implied probability, statistical divergence, prediction markets

## Abstract

We propose a novel approach of model selection for probability estimates that may be applied in time evolving setting. Specifically, we show that any discrepancy between different probability estimates opens a possibility to compare them by trading on a hypothetical betting market that trades probabilities. We describe the mechanism of such a market, where agents maximize some utility function which determines the optimal trading volume for given odds. This procedure produces supply and demand functions, that determine the size of the bet as a function of a trading probability. These functions are closed form for the choice of logarithmic and exponential utility functions. Having two probability estimates and the corresponding supply and demand functions, the trade matching these estimates happens at the intersection of the supply and demand functions. We show that an agent using correct probabilities will realize a profit in expectation when trading against any other set of probabilities. The expected profit realized by the correct view of the market probabilities can be used as a measure of information in terms of statistical divergence.

## 1. Introduction

Measuring the quality of the fit of a model to the data is a central question in statistics. Standard models for probabilistic estimates based on several explanatory variables include the logit and probit models, where the optimal fit is found using maximum likelihood methods. Adding more explanatory variables gives a better fit, but the tradeoff is that the system may become over parametrized and the added predictive power can be limited for variables with small statistical significance. One approach for model selection relies on the use of the Akaike information criterion [1], which penalizes the addition of every explanatory variable, thus finding an optimal balance by selecting only the variables that improve the quality of the model. An up to date overview of model selection based on information-theoretic approaches in the analysis of empirical data can be found Burnham and Anderson [2] or in Guo et al. [3]. However, analysis of large data sets may be best performed by more complex nonlinear models outside of the traditional framework of the regression analysis. For instance, techniques used in machine learning may not directly yield any measure representing the statistical quality of the model in the sense of the Akaike. In such cases, one can evaluate the quality of the model by using the proper scoring rules, such as the Brier score or the log loss.

The quality assessment of a probabilistic estimate of an outcome is traditionally done by a scoring rule. The proper scoring rule compares the probabilistic estimates with the actual outcomes. Different types of scoring have been extensively studied in the previous literature dating back to the 1950’s. Brier [4] introduced a quadratic score function (now called the Brier score) and Good [5] introduced a logarithmic scoring rule. They both represent a proper scoring rule, which gives the highest expected reward to the true probability distribution. This means that the use of a proper scoring rule encourages the forecaster to be honest and maximize the expected reward. There is extensive subsequent work that focused on various extensions, theoretical properties of the scoring rules and on the mathematical representation of such rules. The main results can be found in papers of Savage [6], Matheson and Winkler [7], Schervish [8], or Gneiting and Raftery [9].

Scoring rules compare one probability estimate with one-time observation. However, it is not clear how to measure the quality of the probabilistic estimate if such an estimate evolves in time. Many practical applications (weather predictions, elections, game probabilities, financial estimates) typically involve time varying estimates. The problem of dynamically evolving probability estimates is that any new information that updates such probabilities comes itself at random times, which prevents a natural time grouping of various estimates required for a quality assessment. In addition, a small initial difference in two models may lead to a long-lasting discrepancy that came from a single source if no new information arrives in the meantime, and thus adding (or equivalently averaging) scoring rules over time would disproportionally amplify such differences that should count only once. This means that a correct approach to measuring the quality of probability time series must adequately account for only newly arriving information and isolate its impact on the current estimate.

Our paper gives an entirely new approach to measure the quality of the fit obtained from several alternative probabilistic models that can be used in any setting. The idea is to compare the performance of two models against each other directly, so it gives a pairwise comparison which makes it distinct from the techniques of the proper scoring rules. This approach generalizes the concept of divergence in statistics (such as Kullback-Leibler [10] later studied by Eguchi and Copas [11]) that compares two probabilistic distributions to the time varying setting. Having two different probability estimates opens a possibility of setting a bet that would make the respective agents optimal in the sense of improving their utility functions. The problem of determination of the optimal size of the bet of an agent with true odds against an agent with biased odds was already studied in Kelly [12], but his study was limited to a static setup with fixed odds, one time observation and a logarithmic utility function. We substantially expand this idea and develop an approach that can be applied to a dynamic setting when the probabilistic estimates are evolving in time.

This technique is relatively simple and straightforward. If we have two alternative probability estimates, the discrepancy can be traded off on a hypothetical betting market. The trading mechanism is based on a utility maximization of the two agents. This mechanism finds both the trading price (probability where the two agents agree to trade) and the corresponding volume of such a bet. We show that an agent using the true probabilities has a non-negative expectation of the profit resulting from trading against any other probability estimate, meaning that the true model cannot be beaten in the long run. The only condition for this result to hold is that the agent with the true probabilities uses an increasing concave utility function. In particular, it does not matter what strategy is used by the second agent. When the two agents agree on a matching algorithm, such as the one based on the equilibrium, the resulting expected profit can be used as a definition of divergence of two probabilistic distributions in the sense of information theory in a dynamic setting. The resulting profit from this trading algorithm can be viewed as information gained from the comparison of two probabilistic models.

Our approach can also be applied in situations when there is no explicit model for the resulting probability estimates in terms of some explanatory variables. This happens for instance in cases when the probabilities are quoted by external subjects, such as in the case of weather predictions where we see only the final probabilities of some events such as rain or snow. In such situations, we have no way to assess the quality of the estimates by Akaike’s criterion, but we still can use our approach in terms of the direct comparison of two estimates. Given that we have several models, one cannot statistically identify if the best model corresponds to the true model, which is similar to Akaike’s criterion. Interestingly, our approach can statistically conclude that all studied models are incorrect. That follows from the fact that the expected utility of the trading exposures should be an increasing function of time for the true model and it is possible that this condition is not satisfied for any studied model.

The idea of comparing two probabilities by market trading is novel and only limited related literature exists. Some relevant work has been done in the study of prediction markets that trade probabilities. Wolfers and Zitzewitz [13] give an overview of prediction market types, and their follow up work Wolfers and Zitzewitz [14] determines the size of the optimal position for the agent that maximizes logarithmic utility function for a number of option binary contracts. Arrow et al. [15] argue that prediction markets can help to produce forecasts of event outcomes with a lower prediction error than conventional forecasting methods. Other works focus on checking the quality of probability estimates in different markets using empirical evidence. Ait-Sahalia et al. [16] studied the question whether option markets correctly price the probabilities of movement of the underlying asset. Spann and Skiera [17] compared the forecast accuracy of different methods, namely prediction markets, tipsters and betting odds for soccer games in a static setup. Croxson and Reade [18] studied the efficiency in betting markets after arrival of major news, such as a goal. Forsythe et al. [19] analyzed the Iowa Political Stock Market to ascertain how well markets work as aggregators of information. Rothschild [20] compared the quality of election forecasts from prediction markets and from polls by analysis of the respective probit models. A recent paper of Taleb [21] that studies the evolution of election predictions notes that the resulting probabilistic time series must be a martingale and he argues that the historical time series should have exhibited a fit to a martingale model based on no-arbitrage arguments.

The structure of the paper is as follows. First, we describe the optimal trading behavior of an agent that uses specific probability estimates. This itself is interesting as the maximization of the utility of this agent leads to explicit supply and demand functions for the choice of logarithmic and exponential utility functions. Supply and demand functions are fundamental concepts in economics. However, to the best of our knowledge, there are no known examples of explicit supply and demand functions that are results of the maximization of a utility function. We show that the expected profit for an agent that uses the true probability measure is non-negative regardless of the trading strategy of the rest of the market that match the quotes of the first agent. This result is analogous to a property of a proper scoring rule.

If we have two agents with competing probability models, we also need to describe the matching algorithm that would make the size of the bets acceptable for both of them. The natural choice of the trading price is the point where the supply/demand functions of both agents intersect at an equilibrium point, giving us both the trading price and the trading volume of this match. This itself creates profit-loss statistics from the comparison of the two models, including the statistical significance that measures whether one model is better than a second model. The resulting expected profit can be used as a definition of a statistical divergence in a dynamic setting. We summarize the matching algorithm so that the reader can implement it. The trading on the probability market leads to another novel concept, namely there is a price corresponding to the probability at which the agent is unwilling to trade, meaning that his supply/demand function is equal to zero. Intuitively, the agent should not be willing to change his positions if the quoted price equals his own estimate of the probability, but the agent may have uneven exposures and thus the no-trading point may be shifted. We call this point an implied probability and discuss it in a separate section. We conclude the paper with an illustrative simulated example that compares risk neutral probabilities from the Black-Scholes model [22] with a true volatility 0.25 with models using a low volatility 0.2 and a high volatility 0.3. The true model statistically outperforms both low and high volatility models in less than 100 scenarios. Interestingly, the high volatility model performs slightly better than the low volatility model. The direct comparison of the high volatility model and the low volatility model provides an example of identification of two bad models as neither of the two models has an increasing expected utility function.

## 2. Results

### 2.1. Market Model for Trading Probabilities

Consider a situation when we have two possible binary outcomes, $\left(y_{1},y_{2}\right)$ with $y_{1}+y_{2}=1$. At time *t*, the probability of these outcomes is given by $\left(p_{1}\left(t\right),p_{2}\left(t\right)\right)$. As a process, $p_{1}\left(t\right)$ is the conditional expected value of the final outcome:

$$p_{1}\left(t\right)=E_{t}\left[y_{1}\right]$$

under the true probability measure P. In particular, $p_{1}\left(t\right)$ as a process is a martingale under the true probability measure. The true values $\left(p_{1}\left(t\right),p_{2}\left(t\right)\right)$ are typically unobserved and they are a subject of statistical estimation. Since there can be several competing models for these probabilities, the question is what model is the best among the available alternatives. Let us consider the situation when we have two models that give two probability measures $P^{a}$ and $P^{b}$. Thus we have two probability estimates at time *t*: $p^{a}\left(t\right)=\left(p_{1}^{a}\left(t\right),p_{2}^{a}\left(t\right)\right)$ and $p^{b}\left(t\right)=\left(p_{1}^{b}\left(t\right),p_{2}^{b}\left(t\right)\right)$ of the outcomes $\left(y_{1},y_{2}\right)$. Obviously, $p_{2}^{a}\left(t\right)=1-p_{1}^{a}\left(t\right)$ and $p_{2}^{b}\left(t\right)=1-p_{1}^{b}\left(t\right)$.

Let us limit our analysis to the situation of two outcomes, the generalization to an arbitrary number of the number of outcomes is possible, but the formulas involved become rather complicated which we will address it in subsequent work. Given two different estimates, a natural question is which probability series is better in the sense that it is closer to the true probability. We present an approach to compare these estimators based on the following. When there is any discrepancy between the two estimates $p^{a}\left(t\right)$ and $p^{b}\left(t\right)$ at time *t*, it introduces a possibility of placing a bet. This bet will have two exposures: $N_{1}\left(t\right)$ that corresponds to the win of the first player if the first selection happens and $N_{2}\left(t\right)$ that corresponds to the win of the first player if the second selection happens. These bet sizes will be added to the existing cumulative exposures from the past $\left(V_{1}\left(t\right),V_{2}\left(t\right)\right)$, resulting in

$$V_{1}\left(t+dt\right)=V_{1}\left(t\right)+N_{1}\left(t\right),\;\;V_{2}\left(t+dt\right)=V_{2}\left(t\right)+N_{2}\left(t\right).$$

The market is a zero sum game, and the second agent will collect the negative value of these: $-V_{1}\left(t\right)$ and $-V_{2}\left(t\right)$ respectively. We now discuss how to determine the changes in the exposures $\left(N_{1}\left(t\right),N_{2}\left(t\right)\right)$ and the cumulative exposures $\left(V_{1}\left(t\right),V_{2}\left(t\right)\right)$ that would be acceptable for both agents.

An agent can evaluate the current exposures $V\left(t\right)=(V_{1}\left(t\right),V_{2}\left(t\right))$ by using some utility function U(x) that is increasing and concave. The standard choices are

**Logarithmic** **Utility:** $U\left(x\right)=\log\left(1+\frac{x}{B}\right),$
**Exponential** **Utility:** $U\left(x\right)=1-\exp\left(-\frac{x}{B}\right)$,
**Power** **Utility:** $U\left(x\right)=\frac{{\left(1+\frac{x}{B}\right)}^{1-a}-1}{1-a},a>0.$

The parameter *B* is a free parameter and it can be interpreted as a bankroll. In the case of logarithmic utility, the exposures cannot fall below −B as the evaluation of such a position would be negative infinity, thus bounding the resulting exposures. This is not the case for the exponential utility, where exposures below −B are evaluated with a finite value, but such positions are still evaluated highly negatively, meaning that exposures below −B are possible, but unlikely to be realized. Note that logarithmic utility is a limiting case of a power utility when a→1. As it turns out, the choice of power utility does not lead to analytical formulas for the below described trading algorithm, and thus we will give formulas only for the logarithmic and exponential utilities.

The agent with probability estimates $p^{a}\left(t\right)$ that correspond to probability measure $P^{a}$ assigns an evaluation of his positions $V\left(t\right)=(V_{1}\left(t\right),V_{2}\left(t\right))$ as

$$u^{a}\left(V_{1}\left(t\right),V_{2}\left(t\right)\right)=E^{a}U\left(V\left(t\right)\right)=p_{1}^{a}\left(t\right)\cdot U\left(V_{1}\left(t\right)\right)+p_{2}^{a}\left(t\right)\cdot U\left(V_{2}\left(t\right)\right).$$

Similar evaluation of V(t) appears in Kelly [12] and more recently in Wolfers and Zitzewitz [14], but these works are limited to the logarithmic utility function and to static positions. Note that the evaluation of the trading positions V(t), $u^{a}\left(V_{1}\left(t\right),V_{2}\left(t\right)\right)$, is based on a subjective probability measure $P^{a}$. For any given odds of the first selection that corresponds to the inverse of the traded probability *p*, an agent can add or subtract some quantity from the existing exposures which will result in a more favorable utility value. This creates a supply/demand function for each agent, which represents a volume that the agent is willing to bet given the available market odds $\frac{1}{p}$.

More specifically, if the first agent accepts an additional bet of N(t) units on the first selection at price *p*, his exposures will change to

$$V_{1}\left(t+dt\right)=V_{1}\left(t\right)-\left(\frac{1}{p}-1\right)\cdot N\left(t\right),\;\;V_{2}\left(t+dt\right)=V_{2}\left(t\right)+N\left(t\right),$$

so the changes in the exposures are given by

$$N_{1}\left(t\right)=-\left(\frac{1}{p}-1\right)\cdot N\left(t\right),\;\;N_{2}\left(t\right)=N\left(t\right).$$

If the first selection materializes, the first agent is obliged to pay off $(\frac{1}{p}-1)\cdot N$ units that correspond to the bet size *N* at the $\frac{1}{p}$ odds. If the first selection does not materialize, the agent that accepted the bet will collect the original bet size *N*. Thus for every probability *p* that represents the trading price, we can find the corresponding *N* that maximizes the utility function, thus creating a supply/demand function. The reader should note that the parameter *p* plays a dual role of representing both the trading price and the probability of the first selection. In the following text, parameter *p* is used exclusively for the trading price as quoted by the market, while parameters $p^{a}$ and $p^{b}$ represent subjective probabilities of the respective agents.

The optimal betting size *N* maximizes

$$p_{1}^{a}\left(t\right)\cdot U\left(V_{1}\left(t\right)-\left(\frac{1}{p}-1\right)\cdot N\right)+p_{2}^{a}\left(t\right)\cdot U\left(V_{2}\left(t\right)+N\right).$$

This defines the supply function:

**Definition** **1.** *A supply function $S(p_{1}^{a},V_{1},V_{2},p)$ is equal to N that maximizes*

(1) $$p_{1}^{a}\left(t\right)\cdot U\left(V_{1}\left(t\right)-\left(\frac{1}{p}-1\right)\cdot N\right)+p_{2}^{a}\left(t\right)\cdot U\left(V_{2}\left(t\right)+N\right).$$

*for a given utility function U.*

Given a utility function *U*, the current exposures $V=(V_{1},V_{2})$ and the opinion about the probability $p_{1}^{a}$, the agent finds an optimal bet size for any quoted price *p*. This is how we interpret the supply function.

The following theorem assures that the supply function is well defined:

**Theorem** **1.** *The supply function $S(p_{1}^{a},V_{1},V_{2},p)$ is uniquely defined.*

**Proof** **of Theorem 1.** This is a simple consequence of concavity of the utility function *U* and the proof can end here. For more details, consider the case when *U* has a second derivative: $U^{\prime \prime }\left(x\right)<0$. Define

$$f\left(N\right)=p_{1}^{a}\left(t\right)\cdot U\left(V_{1}\left(t\right)-\left(\frac{1}{p}-1\right)\cdot N\right)+p_{2}^{a}\left(t\right)\cdot U\left(V_{2}\left(t\right)+N\right).$$

The second derivative of f(N) is

$$f^{\prime \prime }\left(N\right)={\left(\frac{1}{p}-1\right)}^{2}p_{1}^{a}\left(t\right)\cdot U^{\prime \prime }\left(V_{1}\left(t\right)-\left(\frac{1}{p}-1\right)\cdot N\right)+p_{2}^{a}\left(t\right)\cdot U^{\prime \prime }\left(V_{2}\left(t\right)+N\right)<0,$$

so *f* is also concave and thus attains a unique maximum. □

The supply function $S(p_{1}^{a},V_{1},V_{2},p)$ gives the size of the bet *N* on the second selection. This is directly the change of the size of the second selection $N_{2}$:

$$N_{2}=S\left(p_{1}^{a},V_{1},V_{2},p\right).$$

The change in the first selection $N_{1}$ is given by

$$N_{1}=-\left(\frac{1}{p}-1\right)\cdot N_{2}=-\left(\frac{1}{p}-1\right)\cdot S\left(p_{1}^{a},V_{1},V_{2},p\right).$$

However, the betting positions can be flipped from $\left(V_{1},V_{2}\right)$ with probabilities $\left(p_{a}^{1},p_{a}^{2}\right)$ to $\left(V_{2},V_{1}\right)$ with probabilities $\left(p_{a}^{2},p_{a}^{1}\right)$. This also flips $N_{1}$ for $N_{2}$, meaning they can be directly determined from the same supply function *S*, but with flipped positions:

$$N_{1}=S\left(1-p_{1}^{a},V_{2},V_{1},1-p\right).$$

Finding the maximum in Equation (1) may not lead in general to a simple closed form formula. In particular, a supply function is not closed form for power utilities even for the simplest choices of the coefficient *a*. On the other hand, the choice of logarithmic or exponential utility functions leads to convenient formulas. We can take the derivative of Equation (1) and find where it equals zero, which is equivalent to

(2) $$\left(\frac{1}{p}-1\right)p_{1}^{a}\left(t\right)\cdot U^{\prime }\left(V_{1}\left(t\right)-\left(\frac{1}{p}-1\right)\cdot N\right)=p_{2}^{a}\left(t\right)\cdot U^{\prime }\left(V_{2}\left(t\right)+N\right).$$

For the choice of the logarithmic utility function, we find

(3) $$S^{log}\left(p_{1}^{a},V_{1},V_{2},B,p\right)=\frac{B\left(p_{1}^{a}-p\right)+p\left(p_{1}^{a}V_{1}-p_{1}^{a}V_{2}-V_{1}\right)+p_{1}^{a}V_{2}}{p-1}.$$

Similarly, exponential utility gives:

(4) $$S^{exp}\left(p_{1}^{a},V_{1},V_{2},B,p\right)=-Bp\log\left(\frac{\left(1-p\right)p_{1}^{a}}{(1-p_{1}^{a})p}\right)+pV_{1}-pV_{2}.$$

We are adding the bankroll *B* as a parameter since the utility functions depend on it. To the best of our knowledge, there are no previous results in the literature that produce analytical formula for a supply function which is a result of utility maximization.

In the later text, we will specify the market behavior of the second agent, leading to an automated procedure of market matching. However, the following result is true regardless of the matching behavior of the second agent. This theorem is analogous to the concept of a proper scoring rule, which means that the true probability estimate cannot be beaten in expectation when compared with any other alternative model. In addition, it does not matter what utility function is used, the true model is expected to generate a profit regardless of the utility used. Thus the exact choice of the utility function is secondary.

**Theorem** **2** (True probability has a non-negative expectation)**.** *The exposures V(t) of an agent trading with the true probability measure P, $p\left(t\right)=(p_{1}\left(t\right),p_{2}\left(t\right))$, satisfy the following inequalities:*

(5) $$U^{-1}\left(E\left[U\left(V\left(s\right)\right)\right]\right)\leq U^{-1}\left(E\left[U\left(V\left(t\right)\right)\right]\right)\leq E\left[V\left(t\right)\right].$$

*In particular, E[V(t)]≥0 for all t≥0.*

**Proof** **of Theorem 2.** This is consequence of a submartingale property of the expected utility and concavity of the utility function. First, note that under the true measure, the process E[U(V(t))] is a submartingale, meaning

$$E\left[U\left(V\left(s\right)\right)\right]\leq E_{s}\left[E\left[U\left(V\left(t\right)\right)\right]\right]$$

for s≤t. This is by design. The expected utility moves either by trading actions of the trader or by the evolution of the probabilities. The trading action of the agent always improves the value of the utility with respect to its probability measure, which is true even for agents with other probability estimates. However, in contrast to other probability estimates, the evolution of true probabilities has a neutral impact on the expected utility as the probability *p* is a martingale under the true probability measure P. Note that other probability estimates $p^{b}\left(t\right)$ are not martingales under the true measure P and the evolution of the probabilities does not have a neutral impact on the conditional expected value, meaning other probabilities are not guaranteed to lead to submartingale evolution.

Second, due to the Jensen’s inequality, we have

E[U(V(t))]≤U(E[V(t)]).

Combining the two properties, we get

E[U(V(s))]≤E[U(V(t))]≤U(E[(V(t))]),

and the inequality stated in the theorem is a result of taking the inverse of the utility function $U^{-1}$, which is increasing. In particular, when s=0, we have V(0)=(0,0) and we find

$$E\left[V\left(t\right)\right]\geq U^{-1}\left(E\left[U\left(V\left(0\right)\right)\right]\right)=U^{-1}\left(U\left(0\right)\right)=0.$$

This finishes the proof. □

Note that while the expected utility process, E[U(V(t))], is a submartingale with an increasing expected value, the same is not true for the expected profit, E[V(t)]. Theorem 2 gives lower bounds for the expected profit, but this does not guarantee that the expected profit is non-decreasing in time. An agent maximizing the utility function may prefer a position with a smaller expectation and a smaller variance over a position with a higher expectation and a higher variance. As an illustrative example, consider an agent with a utility function U(x)=log(1+x) that corresponds to the logarithmic utility function with a choice of parameter B=1. Consider the situation when the true probability is $p=\frac{1}{2}$ and the agent uses this probability for trading. The supply function is positive for probabilities above 0.5, and negative for probabilities below 0.5 with the intercept at 0.5. Consider a second agent asking for a trade at $p^{t}\left(1\right)=0.55$. As we discuss in the later text, this trading price would be chosen if the second agent uses the same utility function, but his probabilistic view is $p^{b}\left(1\right)=0.6$. The supply function gives N=0.1111…, leading to exposures

$$V_{1}\left(1\right)=N_{1}\left(0\right)=-0.0909\ldots ,\;V_{2}\left(1\right)=N_{2}\left(0\right)=0.1111\ldots .$$

This leads to a favorable position for the first agent with the expected utility

E[U(V(1))]=0.5×log(1−0.0909…)+0.5×log(1+0.1111…)=0.005025…,

and the expected profit

E[V(1)]=0.5×(−0.0909…)+0.5×0.1111=0.0101….

**Figure 1:**
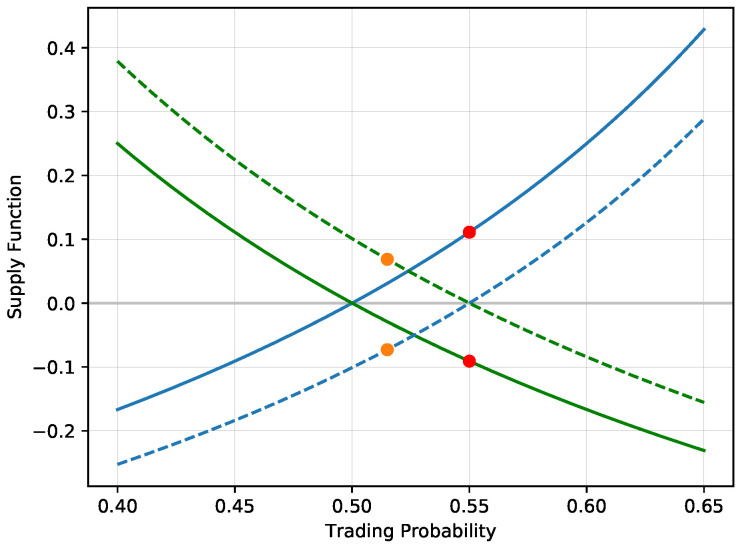
The solid lines represent the supply functions corresponding to the exposure $N_{2}$ for both positions $\left(N_{1},N_{2}\right)$ in green and in blue respectively as a function of the trading price *p*. Note that for $p=\frac{1}{2}$, the agent’s optimal bet size is simply (0,0), the agent is not willing to trade if the odds correspond to the true probability. The first trade happens at odds 0.55 for $(N_{1}\left(0\right),N_{2}\left(0\right))=\left(-0.091,0.111\right)$ (red dots). This shifts the supply function to the dashed lines. Note that the no trading point (0,0) shifts to probability 0.55 as a result of a trade on that price, which will be discussed in the section on implied probability. This means that the sign of the trade changes at the interval [0.5,0.55], resulting in a loss of the expected value. We show the second trade at price 0.515 (orange dots at $(N_{1}\left(1\right),N_{2}\left(1\right))=\left(0.069,-0.073\right)$).

After this trade, the supply function of the first agent shifts down, the supply function is positive for probabilities above 0.55 and negative below 0.55. The first agent accepts additional bets on the first selection only for prices above 0.55. This trade itself is a good illustration of the entire trading procedure. The true probability $p=\frac{1}{2}$ corresponds to the situation of a coin toss with odds $\frac{1}{p}=2$. At these odds, the agent is unwilling to trade. However, at odds equal to $\frac{1}{0.55}=1.8181$, he is willing to accept the bet, creating the exposures $\left(V_{1},V_{2}\right)=\left(-0.0909,0.1111\right)$. After this trade, the agent would accept additional bets only above the price 0.55, or equivalently, for odds below 1.8181. Although accepting bet prices in the interval [0.5,0.55] would further increase his expected profit, it would reduce the expected utility. The first agent would rather reduce his exposure on the first selection at the expense of the expected value. He has already sold the first selection at the higher price of 0.55, so any trade at a buying price below 0.55 would reduce variability of the payoff, which would be reflected in a higher expected utility. Figure 1 shows the supply/demand functions corresponding to the changes of both exposures $\left(N_{1},N_{2}\right)$ before and after the trade, illustrating the shift of the offered volume.

Consider a follow up trade on a price $p^{t}\left(2\right)=0.515$. The existing exposures update to

$$V_{1}\left(2\right)=V_{1}\left(1\right)+N_{1}\left(1\right)=V_{1}\left(1\right)+0.06864\ldots =-0.02226\ldots ,$$

$$V_{2}\left(2\right)=V_{2}\left(1\right)+N_{2}\left(1\right)=V_{2}\left(1\right)-0.07289\ldots =0.03821\ldots ,$$

leading to

E[U(V(2))]=0.5×log(1−0.02226…)+0.5×log(1+0.03821…)=0.00749…,

and

E[V(2)]=0.5×(−0.02226…)+0.5×0.03821…=0.00797…

Thus the expected utility has increased, but the expected value has decreased. Theorem 2 gives a lower bound for the expected value. The inverse of the utility function $U\left(x\right)=\log\left(1+\frac{x}{B}\right)$ is given by

$$U^{-1}\left(x\right)=B\cdot \left(e^{x}-1\right)\geq B\cdot x,$$

which guarantees that the expected value will be in any case at least *B* times the expected utility value (set x=U(E[V(t)])). When B=1, the expected profit is higher than the expected utility. While the expected utility is monotonically increasing, the expected profit may decrease as illustrated by the above example.

Theorem 2 is a key result for model comparison. The expected profit for the model with true probabilities is positive against any other alternative model. This result does not depend on the choice of the utility function, all that is needed here is that the utility function is increasing and concave. It also does not depend on the potential trading strategy of the second agent. In particular, the above described betting strategy with true probabilities is expected to generate a positive profit against the rest of the market even with multiple actors in the market.

### 2.2. Market Matching for Two Models

If we have two models to compare, the question is how to set the market matching algorithm for both agents representing the two alternative probabilistic views. While the results of the previous sections are valid regardless of the actions of the remaining market actors, this section focuses on a trading algorithm for two agents, where the trading price and the volume is acceptable for both sides. Let us assume that the second agent is also maximizing a utility function, but since he uses a different set of probabilities, the valuation of his exposures is different. The exposures of the second agent are $\left(-V_{1},-V_{2}\right)$ as he is on a negative side of the trade. Thus both agents have a supply/demand function based on how much they are willing to bet, and it is natural to set the bet size *N* where the two functions intersect at the equilibrium. As discussed in the previous text, the trading behavior of the second agent is irrelevant for the profitability of an agent that uses true probabilities. For sake of symmetry, it makes sense that both agents use the same utility function. The supply function for the second agent must be taken with a negative sign as he is filling a negative position −V.

A general approach to a matching algorithm requires one to determine the trading price and the trading volume where the two supply and demand functions for the two agents intersect. The trading price $p^{t}$ solves

(6) $$S\left(p_{1}^{a},V_{1},V_{2},p^{t}\right)=-S\left(p_{1}^{b},-V_{1},-V_{2},p^{t}\right),$$

which is a point on the *x*-axis where the supply of the first agent with probabilistic view $p_{1}^{a}$ and exposures $V_{1}$ and $V_{2}$ matches the negative supply of the second agent with probabilistic view $p_{1}^{b}$ and exposures $-V_{1}$ and $-V_{2}$. Similarly, the exact volume *N* to trade is given by the supply function itself

(7) $$N=S(p_{1}^{a},V_{1},V_{2},p^{t}).$$

Since we do not have a supply function for an arbitrary utility function, we will limit ourselves to the situations of the logarithmic and exponential utility functions. It does not matter for the statistical comparison of different probability models as the true model will always outperform any other model regardless of the choice of the utility function. The trading price for the logarithmic utility function is given by

(8) $$p^{t,log}=\frac{Bp_{1}^{a}+Bp_{1}^{b}+p_{1}^{a}V_{2}-p_{1}^{b}V_{2}}{2B-p_{1}^{a}V_{1}+p_{1}^{b}V_{1}+p_{1}^{a}V_{2}-p_{1}^{b}V_{2}},$$

with the corresponding volume

(9) $$N^{log}\left(p_{1}^{a},p_{1}^{b},V_{1},V_{2},B\right)=\frac{B^{2}\left(p_{1}^{a}-p_{1}^{b}\right)+B\left(p_{1}^{a}\left(2p_{1}^{b}-1\right)-p_{1}^{b}\right)\left(V_{1}-V_{2}\right)+V_{1}V_{2}\left(p_{1}^{b}-p_{1}^{a}\right)}{B\left(p_{1}^{a}+p_{1}^{b}-2\right)+V_{1}\left(p_{1}^{a}-p_{1}^{b}\right)}.$$

Note that the initial values when $V_{1}=V_{2}=0$ simplify to

$$p^{t,log}=\frac{p_{1}^{a}+p_{1}^{b}}{2},$$

meaning that the trading price is at the arithmetic average of the two probability estimates and the corresponding exposures are

$$N_{1}=B\cdot \frac{p_{1}^{a}-p_{1}^{b}}{p_{1}^{a}+p_{1}^{b}}=B\cdot \frac{\frac{p_{1}^{a}}{p_{1}^{b}}-1}{\frac{p_{1}^{a}}{p_{1}^{b}}+1},\;\;N_{2}=B\cdot \frac{p_{2}^{a}-p_{2}^{b}}{p_{2}^{a}+p_{2}^{b}}=B\cdot \frac{\frac{p_{2}^{a}}{p_{2}^{b}}-1}{\frac{p_{2}^{a}}{p_{2}^{b}}+1}.$$

This corresponds to the model comparison of two static models where we have only one reading, and where one can use the traditional scoring function.

The trading price for the exponential utility function is given by

(10) pt,exp=p1ap1bp1bp1b1−p1ap1b1−p1bp1b+p1ap1bp1bp1b.

and the volume is

(11) Nexp(p1a,p1b,V1,V2,B)=p1ap1bp1bp1b−Blogp1ap1b1−p1b1−p1ap1bp1b+V1−V21−p1ap1b1−p1bp1b+p1ap1bp1bp1b.

In contrast to the logarithmic utility, the trading price for the exponential utility model does not depend on the existing exposures $\left(V_{1},V_{2}\right)$. The trading price is a scaled geometric average of the two probability estimates.

**Remark** **1** (Relationship to statistical divergence)**.** *The resulting expected profit E[V(T)] from the above described matching algorithm based on utility maximization can be used as a definition of statistical divergence D between two probability measures P and Q as*

$$D\left(P||Q\right)=E^{P}\left[V\left(T\right)\right]\geq 0,$$

*where the P is the true measure based on Theorem 2. Moreover, the only way that the expected profit E[V(T)] is exactly zero is when there is no trade, meaning that the two probabilistic measures much be identical P=Q, which is a condition required for statistical divergence. The important generalization is that this divergence is defined for time evolving probabilities.*

*For instance, consider two probabilistic measures $P=P^{a}$, $Q=P^{b}$ and a logarithmic matching algorithm with B=1 in a static case. We have seen that the resulting exposures ($p=p^{a},q=p^{b}$) are*

$$V_{1}=N_{1}=\frac{\frac{p_{1}}{q_{1}}-1}{\frac{p_{1}}{q_{1}}+1},\;\;V_{2}=N_{2}=\frac{\frac{p_{2}}{q_{2}}-1}{\frac{p_{2}}{q_{2}}+1},$$

*so the expected profit under the true measure P is given by*

$$\begin{array}{ccc} E^{P}\left[V\right] & = & p_{1}\cdot \frac{\frac{p_{1}}{q_{1}}-1}{\frac{p_{1}}{q_{1}}+1}+p_{2}\cdot \frac{\frac{p_{2}}{q_{2}}-1}{\frac{p_{2}}{q_{2}}+1}\\ & = & \frac{p_{1}}{q_{1}}\cdot \frac{\frac{p_{1}}{q_{1}}-1}{\frac{p_{1}}{q_{1}}+1}\cdot q_{1}+\frac{p_{2}}{q_{2}}\cdot \frac{\frac{p_{2}}{q_{2}}-1}{\frac{p_{2}}{q_{2}}+1}\cdot q_{2}\\ & = & \int_{\Omega }f\left(\frac{dP}{dQ}\right)dQ \end{array}$$

*for function*

$$f\left(x\right)=\frac{x^{2}-x}{x+1}.$$

*This is a type of f-divergence.*

*The divergence which is a result of maximization of the exponential utility function is not an f-divergence. However, the exponential utility plays a role in the Kullback-Leibler divergence. The Kullback-Leibler divergence can be viewed as a bet with resulting exposures*

$$V_{1,KL}=N_{1,KL}=\log\left(p_{1}\right)-\log\left(q_{1}\right),\;V_{2,KL}=N_{2,KL}=\log\left(p_{2}\right)-\log\left(q_{2}\right).$$

*After such a trade, both agents would keep zero exponential utility with B=1 as*

$$\begin{array}{ccc} E^{P}\left[U\left(V\right)\right] & = & p_{1}\cdot \left(1-\exp\left(-V_{1,KL}\right)\right)+p_{2}\cdot \left(1-\exp\left(-V_{2,KL}\right)\right)\\ & = & p_{1}\cdot \left(1-\frac{q_{1}}{p_{1}}\right)+p_{2}\cdot \left(1-\frac{q_{2}}{p_{2}}\right)=0, \end{array}$$

*and*

$$\begin{array}{ccc} E^{Q}\left[U\left(-V\right)\right] & = & q_{1}\cdot \left(1-\exp\left(V_{1,KL}\right)\right)+q_{2}\cdot \left(1-\exp\left(V_{2,KL}\right)\right)\\ & = & q_{1}\cdot \left(1-\frac{p_{1}}{q_{1}}\right)+q_{2}\cdot \left(1-\frac{p_{2}}{q_{2}}\right)=0. \end{array}$$

*This means that in a static setting, the exposures from the Kullback-Leibler leave both agents ambivalent with a situation of no trade. Since the expected exponential utility function has an approximately parabolic shape, the maximum is achieved close to the midpoints of the two zeroes, namely at $0.5\times N_{1,KL}$ and $0.5\times N_{2,KL}$. In particular, the initial exposure $N_{2}$ satisfies*

p1p1bq1p1b−logp1p1b1−q1p1b1−p1p1bq1p1b1−p1p1b1−q1p1b+p1p1bq1p1b≈12×log1−p11−q1,

*which is true with a remarkable precision.*

*One can define a statistical divergence in a dynamic setting by adding the trading positions from the divergence in a static setting. For the case of Kullback-Leibler divergence, we can define*

$$\begin{array}{ccc} V_{1}\left(t+dt\right) & = & V_{1}\left(t\right)+N_{1}\left(t\right)=V_{1}\left(t\right)+\left(\log\left(p_{1}\left(t\right)\right)-\log\left(q_{1}\left(t\right)\right)\right),\\ V_{2}\left(t+dt\right) & = & V_{2}\left(t\right)+N_{2}\left(t\right)=V_{2}\left(t\right)+\left(\log\left(p_{2}\left(t\right)\right)-\log\left(q_{2}\left(t\right)\right)\right). \end{array}$$

*This is a natural construction, which moreover guarantees that the process E[V(t)] is non-decreasing under the true measure. This is a simple consequence of a divergence property as $E^{P}\left[N\left(t\right)\right]\geq 0$ at each time t. However, this construction ignores the information flow in time as any long lasting discrepancy is added repeatedly. As a consequence, methods based on utility maximization that properly reflect the information flow in time turn out to be superior as we illustrate in the final section.*

**Figure 2:**
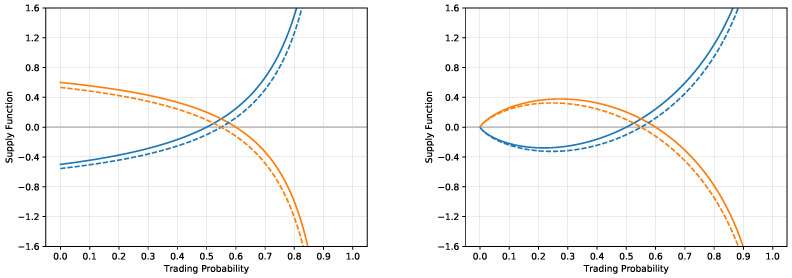
The supply functions corresponding to the exposure $N_{2}$ for an agent with $p_{1}^{a}=0.5$ (solid blue lines) and the demand functions for an agent with $p_{1}^{b}=0.6$ (solid orange lines) for log utility (**left**) and exponential utility (**right**) for B=1 as a function of the trading price *p*. The functions intersect at p=0.55 for the log utility at the level corresponding to N=0.1111… and at p=0.55051… for the exponential utility at the level corresponding to N=0.1116… When the two agents trade off the discrepancy in the probability estimates, their respective supply and demand functions shift to the dashed lines. In particular, the shifted supply and demand functions intersect at the same probability levels as before, but the intersection is now at the level 0, so the discrepancy is fully traded.

Figure 2 illustrates the situation when the two agents trade two probability estimates, one with $p_{1}^{a}=0.5$ and one with $p_{1}^{b}=0.6$ with zero exposures $V_{1}=V_{2}=0$ for both situations of the logarithmic and exponential utility functions. Both agents produce the supply/demand functions. In the situation of the logarithmic utility, the supply/demand functions intersect at the trading price $p^{t,log}=0.55$ with the corresponding volume $N^{log}=0.1111\ldots$ The exponential utility gives slightly different results, the trading price is at $p^{t,exp}=0.55051\ldots$ and the resulting volume is $N^{exp}=0.1116\ldots$

For the comparison statistics, all we need to check is whether the profit statistic,

$$V^{p}\left(t\right)=y_{1}\cdot V_{1}\left(t\right)+y_{2}\cdot V_{2}\left(t\right),$$

is statistically positive. This is the exposure corresponding to the winning side, which is observed only after observing the ultimate outcome in one comparison. If we have *n* scenarios that are independent and identically distributed, the ratio

$$\frac{{\bar{V}}^{p}\left(t\right)-E\left[V\left(t\right)\right]}{\frac{\sigma (V(t))}{\sqrt{n}}}$$

will converge to a standard normal distribution N(0,1) according to the Central Limit Theorem. Since the standard deviation of V(t) is typically not observed, we can introduce

$$S^{2}\left(t\right)=\frac{1}{n-1}\sum_{i=1}^{n}{\left(V_{i}^{p}\left(t\right)-{\bar{V}}^{p}\left(t\right)\right)}^{2},$$

and the test statistic

$$T=\frac{{\bar{V}}^{p}\left(t\right)-E\left[V\left(t\right)\right]}{\frac{S(t)}{\sqrt{n}}}$$

has *t*-distribution with n−1 degrees of freedom. In particular, we can test the hypothesis that E[V(t)]=0, giving the statistical significance to the comparison of two alternative models. The absolute magnitude of ${\bar{V}}^{p}\left(t\right)$ is not important as different utilities may scale this value up or down, but rather the statistical significance depends on its ratio with the corresponding standard deviation.

### 2.3. Implementation of the Matching Algorithm

Let us summarize the algorithm that matches two probability estimates $p^{a}$ and $p^{b}$ needed for the implementation. The procedure is rather simple. The algorithm needs to give the change of the exposures for both $N_{1}\left(t\right)$ and $N_{2}\left(t\right)$, which is given by the function $N(p_{1}^{a},p_{1}^{b},V_{1},V_{2},B)$. Here, one can choose either the function *N* obtained from the logarithmic utility or the function obtained from the exponential utility. Since the expected profit of the true probability is non-negative regardless of the choice of the utility function, it does not matter which one is chosen. We list both of them, but the actual implementation should choose one.

The functions of the matching volume are given by Equation (9) for the logarithmic utility and by Equation (11) for the exponential utility. Next, we need to determine the evolution of the exposures $\left(V_{1}\left(t\right),V_{2}\left(t\right)\right)$ as time series. This is done by setting the initial values:

$$V_{1}\left(0\right)=0,\;\;V_{2}\left(0\right)=0$$

and the updated exposures are given by

(12) $$\begin{array}{ccc} V_{1}\left(t+1\right) & = & V_{1}\left(t\right)+N\left(1-p^{a}{\left(t\right)}_{1},1-p_{1}^{b}\left(t\right),V_{2}\left(t\right),V_{1}\left(t\right),B\right), \end{array}$$

(13) $$\begin{array}{ccc} V_{2}\left(t+1\right) & = & V_{2}\left(t\right)+N\left(p_{1}^{a}\left(t\right),p_{1}^{b}\left(t\right),V_{1}\left(t\right),V_{2}\left(t\right),B\right). \end{array}$$

## 3. Implied Probability

This section applies to an arbitrary number of possible outcomes and thus we present it in the full generality. For a given selection *i*, current exposures $V=(V_{1},\ldots ,V_{n})$, we have a supply function $S(p_{i}^{a},V,p)$ which gives an optimal trading volume for any given price *p*. There is a price $p_{i}^{imp}$ for which the supply function gives exactly zero, meaning that the agent is already at the maximal expected utility and thus unwilling to trade any volume *N* for that price quote. It is natural to call this price an implied probability.

**Definition** **2.** *The implied probability $p_{i}^{imp}$ is a solution of the equation*

(14) $$S(p_{i}^{a},V,p^{imp})=0.$$

**Theorem** **3.** *The implied probability is given by*

(15) $$p_{i}^{imp}=\frac{p_{i}^{a}\cdot U^{\prime }\left(V_{i}\right)}{\sum_{k=1}^{n}p_{k}^{a}\cdot U^{\prime }\left(V_{k}\right)}.$$

**Proof** **of Theorem 3.** We want to find the trading price $p_{i}^{imp}$ for which the volume N=0 maximizes the expected utility after rebalancing the exposures $V=(V_{1},\ldots ,V_{n})$. The *i*-th exposure obliges to pay out $(\frac{1}{p}-1)\cdot N$, so the exposure $V_{i}$ updates to $V_{i}-\left(\frac{1}{p}-1\right)\cdot N$, all the other exposures collect *N*, so they update to $V_{k}+N$. The expected utility of the updated exposures is given by

$$\sum_{k=1,k\neq i}^{n}p_{k}^{a}\cdot U\left(V_{k}+N\right)+p_{i}^{a}\cdot U\left(V_{i}-\left(\frac{1}{p}-1\right)\cdot N\right).$$

Solving for the maximum when N=0 gives after differentiation

$$\sum_{k=1}^{n}p_{k}^{a}\cdot U^{\prime }\left(V_{k}\right)=\frac{p_{i}^{a}\cdot U^{\prime }\left(V_{i}\right)}{p},$$

which gives

$$p_{i}^{imp}=\frac{p_{i}^{a}\cdot U^{\prime }\left(V_{i}\right)}{\sum_{k=1}^{n}p_{k}^{a}\cdot U^{\prime }\left(V_{k}\right)}.$$

 □

**Example** **1.** *The case of the logarithmic utility function gives $U^{\prime }\left(x\right)=\frac{1}{1+\frac{x}{B}}\cdot \frac{1}{B}$, leading to*

$$p_{i}^{imp,log}=\frac{\frac{p_{i}^{a}}{1+\frac{V_{i}}{B}}}{\sum_{k=1}^{n}\frac{p_{k}^{a}}{1+\frac{V_{k}}{B}}}.$$

*When all exposures are zero, we simply retrieve $p_{i}^{imp}=p_{i}^{a}$, meaning that the agent would not engage in any trade when the market quote would agree with his own beliefs. The same is true when all exposures are equal. However, unbalanced exposures would shift the implied probability in an intuitive way. When the market previously bet on exposure i more frequently than on other selections, the agent would end up with a negative exposure $V_{i}<<0$. When all the other exposures are insignificant or even positive, the fraction $\frac{1}{1+\frac{V_{i}}{B}}$ would be greater than 1, thus leading to inequality $p_{i}^{imp}>p_{i}^{a}$. Higher implied probability means that the agent would accept the bet only for prices above $p_{i}^{imp}$, meaning that he would quote lower odds. This makes perfect sense and this procedure describes an algorithmic approach how to adapt the odds to the market conditions. Analogously, when the exposure $V_{i}$ becomes unproportionally positive, the market does not believe in materialization of that selection, leading to smaller implied volatility $p_{i}^{imp}<p_{i}^{a}$.*

*Exponential utility gives $U^{\prime }\left(x\right)=\frac{1}{B}e^{-\frac{x}{B}}$, leading to implied probability*

$$p_{i}^{imp,exp}=\frac{p_{i}^{a}e^{-\frac{V_{i}}{B}}}{\sum_{k=1}^{n}p_{k}^{a}e^{-\frac{V_{k}}{B}}}.$$

*Again, the unbalanced exposures will shift the implied probability in a similar fashion as in the case of logarithmic utility.*

*For the sake of completeness, the power utility function has $U^{\prime }\left(x\right)=\frac{1}{B}{\left(1+\frac{x}{B}\right)}^{-a}$, giving*

$$p_{i}^{imp,pow}=\frac{\frac{p_{i}^{a}}{{\left(1+\frac{V_{i}}{B}\right)}^{a}}}{\sum_{k=1}^{n}\frac{p_{k}^{a}}{{\left(1+\frac{V_{k}}{B}\right)}^{a}}}.$$

*The choice of a=1 corresponds to the logarithmic utility, so the formulas indeed agree.*

## 4. Discussion: Application to the Black-Scholes Model with Correct and Misspecified Volatilities

Let us illustrate how we can apply these techniques for model selection in a time evolving setup. For explanatory purposes, we use simulated data where we generate several competing models where one of them has exact probabilities and the others are misspecified. We choose the Black-Scholes model of the stock price driven by a Brownian motion W(t):

$$S\left(t\right)=S\left(0\right)\exp(\sigma W\left(t\right)-\frac{1}{2}\sigma^{2}t)$$

and a digital option contract that pays off $1 if the terminal value of the stock S(T) exceeds a specified strike price *K*. The price of this digital option is given by

$$\Phi \left(\frac{1}{\sigma \sqrt{T-t}}\cdot \log\left(\frac{S(t)}{K}\right)-\frac{1}{2}\sigma \sqrt{T-t}\right),$$

where *t* is the current time, *T* is the terminal time, S(t) is the price of the stock at time *t* and *K* is the strike. The price of this digital option also depends on a parameter called the volatility σ.

The function Φ(·) is the cumulative distribution function of the normal distribution.

**Figure 3:**
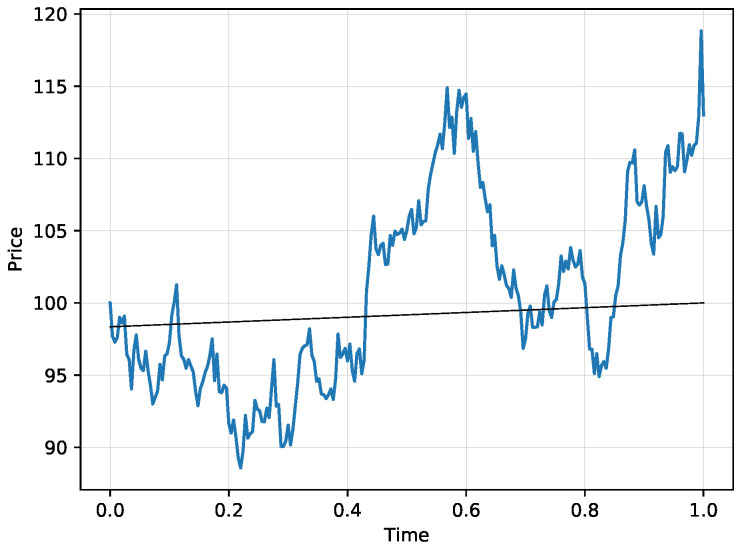
A simulated path of a stock price evolution in the Black-Scholes model with S(0)=100 and the volatility parameter 0.25.

**Figure 4:**
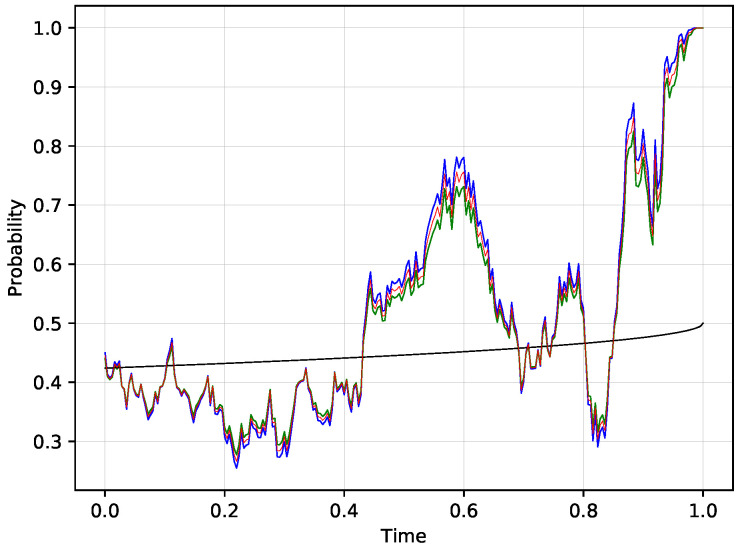
The corresponding prices of a digital option from a model with true volatility parameter (blue), with the high volatility parameter 0.3 (green) and the implied probability path where the two models trade in the equilibrium between the two competing models (red) for the stock evolution scenario from Figure 3. The black curves in both graphs indicate the point where the two models agree on the price of the corresponding digital option, so a close proximity to that curve makes the two models virtually indistinguishable.

Consider the situation when S(0)=100, K=100, T=1 and the true volatility parameter is a constant σ=0.25. Let us study two other competing models, one with a higher volatility 0.3 and one with a smaller volatility 0.2. The frequency of trading is set to $dt=\frac{1}{250}$, which in practice corresponds to one day. Figure 3 shows one scenario of a stock price evolution and Figure 4 shows the prices of the digital option for this scenario corresponding to a model with the true volatility 0.25 (in blue) and to a model with high volatility 0.3 (green). Note that both models lead to very similar probability estimates and the difference is barely visible. Moreover, the two models—one with the correct volatility and one with the high volatility—agree on the price of the digital option on a curve plotted in black, so each time the stock price reaches the black curve, the probabilities from both models are the same. Thus the difference between the two models is marginal when the probabilities are near the black curve, which is visible from the graph. The difference gets more pronounced when the probabilities depart from the indicated black curve. The two models trade off any difference on the price level corresponding to the implied probability indicated by the red curve. We use log utility in this example, the results from the exponential utility are nearly identical.

For each simulated stock price scenario S(t), we generate the corresponding evolution of the exposures $\left(V_{1}\left(t\right),V_{2}\left(t\right)\right)$ by using the algorithm described in the implementation section. Theorem 2 assures that the true model will outperform any other model regardless of the choice of the utility function and for illustration, we choose the logarithmic utility function U(x)=log(1+x), where we pick the parameter B=1. The results based on the two other alternative approaches studied earlier, namely the matching algorithm based on the exponential utility function and the dynamic version of the Kullback-Leibler divergence, are studied at the end of this section for comparison. In order to have a deeper insight about the evolution of the exposures $V_{1}\left(t\right)$ and $V_{2}\left(t\right)$, let us make the following observation. When the two models agree on the probability, any possible trade must happen on the same price and as a result, the implied probability will agree with the estimate from the two models:

$$p_{1}^{a}\left(t\right)=p_{1}^{b}\left(t\right)=p_{1}^{imp}\left(t\right).$$

The implied probability agrees with the original probability only if we have

$$V_{1}\left(t\right)=V_{2}\left(t\right),$$

which is easy to see from the formulas for the implied probability. In our situation, it means that each time the stock price crosses the black line as in Figure 4, the two exposures $V_{1}$ and $V_{2}$ cross at that time. Note that each such cross is at the trading expense of the true model. The reason is that the true model uses a smaller volatility 0.25 in comparison to the high 0.3 volatility model and thus it sees the probability of the additional cross as smaller than that of a model with a higher volatility. However, in the region between the two crosses, the true model sells to the high volatility model the event that the probability will reverse, i.e., it is closer to 1 when above the black curve and closer to 0 when below the black curve in comparison to the high volatility model. Thus when the additional cross happens, the true model realizes a loss. On the other hand, when there is no additional cross, the true model realizes a profit as being closer the ultimate outcome in comparison to the model with high volatility.

**Figure 5:**
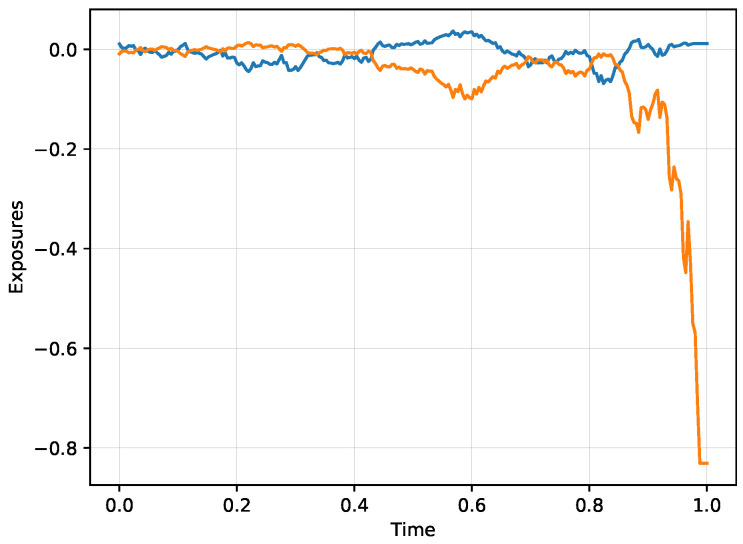
Evolution of $V_{1}$ (blue) and $V_{2}$ (orange) corresponding to the scenario from Figure 4. Note that the two exposures cross each time when the stock price crosses the black curve.

Figure 5 shows the evolution of the exposures $V_{1}$ and $V_{2}$ for the scenario from Figure 4. Note that the exposures cross exactly at the times when the two models agree on the price of the digital option, or equivalently when the stock price crosses the black curve. Each subsequent crossing happens at a lower level since the true model buys the side which is not yet materialized and results in a loss if the probabilities return to the black curve. The most notable time between two visits of the black curve happens in the time interval [0.432,0.692]. The model with the true volatility is on the buy side of the event that the digital option will end up in the money. The true model increases the exposure $V_{1}$ which will materialize if the option ends up in the money at the expense of the exposure $V_{2}$ which will materialize otherwise. This view is obviously correct in terms of the probabilities, but it still leaves a possibility of a random return back to the black curve. This reversal indeed happens at time t=0.692, resulting in a trading loss for the true model at that time. The price process makes a few follow up crosses of the black curve with the last cross at time t=0.852. From that point on, the true model is mostly on the buying side of $V_{1}$ that will ultimately win, improving the previously lost positions. When the model is sure with near certainty about the winning side, it effectively sells out the losing side, creating a relatively large negative exposure which does not materialize. The ultimate win is $V_{1}\left(1\right)=0.012019\ldots$ and thus the true model realizes a profit over the model with high volatility in this particular scenario.

**Figure 6:**
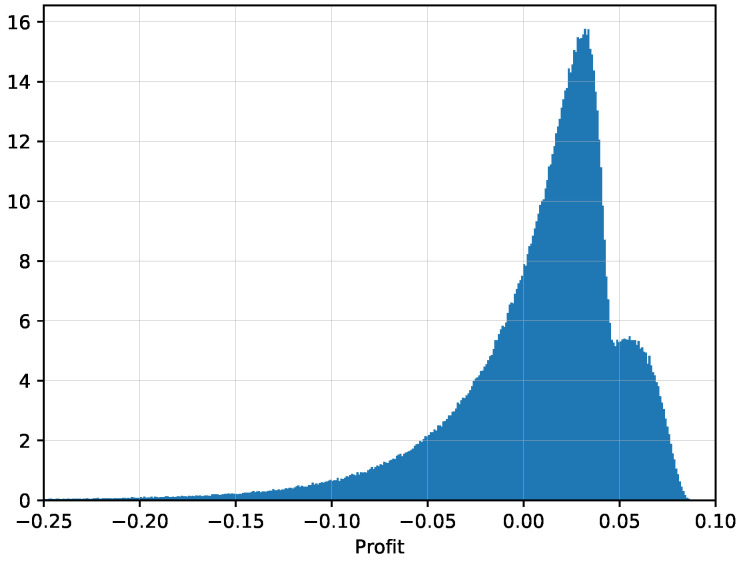
Distribution of the realized profit from comparison of the Black-Scholes model with the true volatility σ=0.25 and the high volatility σ=0.3, number of simulations n=1,000,000.

The above example describes the sources of the profit for both models considered. The true model realizes the profit only after the last visit of the point where both models agree on the price, which is represented by the black curve in Figure 4. The model with high volatility profits from returns to that curve. However, the model with high volatility expects more such visits than what occurs in reality, thus realizing a loss in expectation against the true model. Figure 6 shows a histogram of the realized profit of trading of the true model against the model with high volatility. Since the actual implementation of the model comparison is rather fast, we could have used n=1,000,000 simulations of the price evolutions. The average profit was $\bar{V}=0.00824\ldots$ with a standard deviation of $\sigma_{V}=0.0457\ldots$ Thus the 95% confidence interval for the mean winning is

$$\bar{V}\pm 1.96\times \frac{\sigma_{V}}{\sqrt{n}}\approx 0.00824\pm 0.00009=\left[0.00815,0.00833\right].$$

These values also indicate how many scenarios must be analyzed before being reasonably sure that one model is better than the other. The one sided 95% confidence interval for the average win ${\bar{V}}_{n}$ from *n* observations is given by

$$[\mu -1.645\frac{\sigma }{\sqrt{n}},+\infty ).$$

In order for this interval to be positive valued, the left end point needs to be above zero and the minimal such *n* solves

$$\mu -1.645\frac{\sigma }{\sqrt{n}}=0.$$

The resulting *n* is given by

$$n=\frac{1.645^{2}}{{\left(\frac{\mu }{\sigma }\right)}^{2}},$$

where $\frac{\mu }{\sigma }$ is an information ratio. Our estimated values give n=90, so in this particular situation of comparing the models with true volatility and high volatility, it would take 90 scenarios to have positive ${\bar{V}}_{n}$ statistics with a probability of 95%. Given that the probabilities representing the value of the digital option are close to each other to start with for this choice of volatilities, the number of such scenarios seems very reasonable. The closer the models, the more scenarios one would expect to be required and vice versa.

Let us also take a closer look at the exact shape of the distribution in Figure 6. As the losses of the true model come only from the repeated crossing of the black curve, the size of the loss should be proportional to the number of such crosses. Since the price process is Markov, it has no memory and thus the number of such crosses should be approximately geometric if we consider discrete monitoring, meaning that each additional cross happens only with probability q<1. Thus the left tail of the profit distribution that corresponds to the losses should be approximately geometric (or exponential), which is clearly visible in the graph. On the other hand, the profits are realized when the stock price stays for the most of the remaining time fully above or fully below the black curve, in which case the realized profit reflects the price fluctuations on the way, resulting in a normal distribution. These scenarios represent only a minority of possible situations, but enough to be visible in the right side of the histogram.

After we studied one particular simulation, we can focus on the evolution of the expected profit at time *t*, E[V(t)], and the corresponding evolution of the utility function E[U(V(t))]. We consider n=1,000,000 different simulated scenarios, which results in very precise estimates. We have previously shown in Theorem 2 that the process E[U(V(t))] is a submartingale under the true measure, and thus the expected value should be a non-decreasing function of time. The monotonicity of the process E[V(t)] is not guaranteed, but the specific choice of the utility function guarantees the inequality E[V(t)]≥E[U(V(t))].

**Figure 7:**
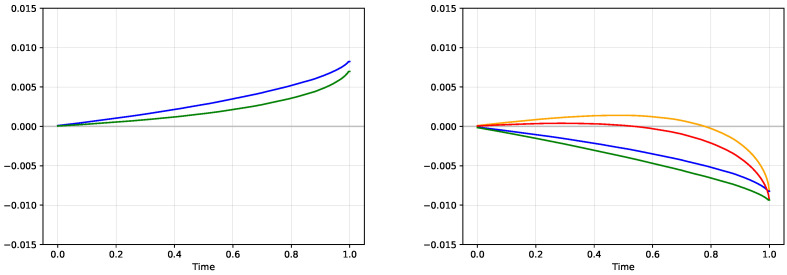
Comparison of the true model and the model with the high volatility. Left: expected profit (blue) and expected utility (green) under true probabilities. Both functions are increasing due to the submartingale property. Right: expected profit (orange) and expected utility of the position −V(t) from the perspective of the probabilities from a model with high volatility. Since this is an incorrect model, we also plot the expected profit under the real measure (blue) and the expected utility under the real measure (green).

We study 3 model comparisons, true model versus the high volatility model, true model versus low volatility model and high volatility model versus low volatility model. The left pane of Figure 7 shows the time evolution of E[U(V(t))] (green) and E[V(t)] (blue) from the comparison of the true model with the high volatility model. The expected utility is clearly increasing and the expected profit dominates the expected utility, confirming the theoretical result that the true model should realize a profit against any other model in expectation. The right pane of the same figure presents the evaluation of the position −V(t) from the perspective of the model with incorrect probabilities. The red graph is the expected utility

$$E^{H}\left[U\left(-V\left(t\right)\right)\right]=p_{1}^{H}\left(t\right)\cdot U\left(-V_{1}\left(t\right)\right)+p_{2}^{H}\left(t\right)\cdot U\left(-V_{2}\left(t\right)\right)$$

from the perspective of probability weights $p^{H}=\left(p_{1}^{H}\left(t\right),p_{2}^{H}\left(t\right)\right)$ that correspond to the value of the digital option with high volatility 0.3. The orange graph corresponds to the expected value $E^{H}\left[-V\left(t\right)\right]$. Initially, the agent with the set of probabilities $p^{H}$ subjectively experiences an increase in both his utility and expectation functions, which is the reason why the agent is willing to trade in the first place, but with increasing time, his position eventually becomes negative even when using his own shifted probabilities. The real profit as measured by the correct probabilities E[−V(t)] is graphed in blue, and it is the negative of the blue line from the left pane. Both models agree on the final payoff V(T) and thus the blue and the orange curves meet at the end. The evolution of the expected utility under the true measure, E[U(−V(t))], is graphed in green.

**Figure 8:**
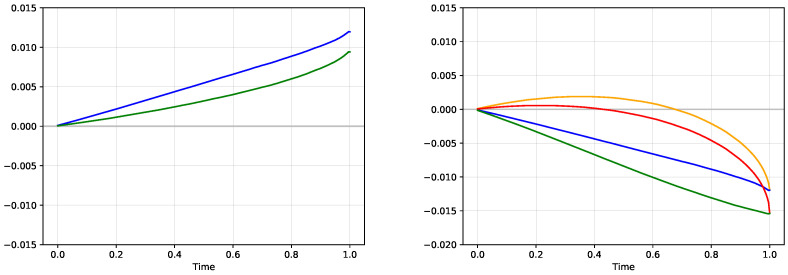
Comparison of the true model and the model with the low volatility analogous to the situation in Figure 7. The left pane shows the expected utility (green) and the expected profit from the perspective of the true model, the right pane shows the evaluations of the position −V(t) both from the perspective of the low volatility model (expected utility in red, expected profit in orange) and from the perspective of the true probabilities (expected utility in green, expected profit in blue).

**Figure 9:**
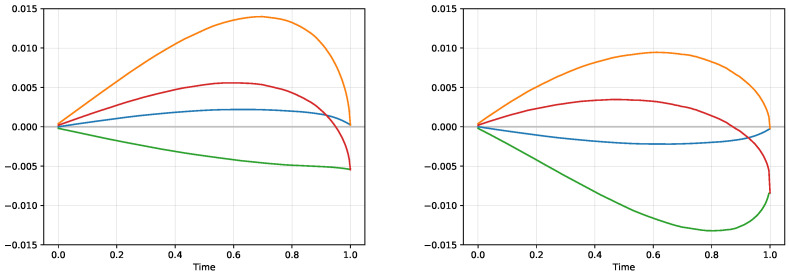
Comparison of two incorrect models, one with high volatility and one with low volatility. Left pane shows the evaluation of the position V(t) from the perspective of the high volatility model (expected utility in red, expected profit in orange) together with the objective valuation of the same position from the perspective of the true model (expected utility in green, expected profit in blue). The right pane shows the evaluation of the position −V(t) both from the perspective of the low volatility model with the analogous graphs.

Figure 8 shows the same analysis for the comparison of the true model and the model with low volatility. The resulting graphs illustrate the same situation when the true model realizes a profit against the low volatility model. In fact, the true model realizes a slightly higher profit when trading against the low volatility model than the high volatility model. This opens a question whether the high volatility model would perform better in direct comparison with the low volatility model. Figure 9 illustrates this situation. Using the subjective probabilities, both models start to increase their respective expected utility functions. But since both models do not represent the correct probabilities, it is not expected that they would keep increasing for the entire time period. In fact, both models start to experience a decrease of their respective expected utilities after the first half of the trading period, which statistically indicates that both models in question are incorrect. This means that a direct comparison of two incorrect models may lead to a conclusion that neither of them is good.

The interesting situation is to observe the evolution of the expected utility and the expected profit under the objective measure, which is available in our simulated experiment. If these probabilities are not available, we can still estimate the expected profit E[V(t)] from the observed average profit ${\bar{V}}^{p}$. As seen from the graph, the expected profit of the high volatility model is always above zero when trading against the low volatility model. However, a substantial part of these gains is given up close to the expiration of the digital contract. Still, the final profit is slightly above zero, indicating that overestimating volatility would lead to better results than underestimating volatility.

Table 1 summarizes the profit-loss *V* statistics together with statistical significance of the results. The true model clearly outperforms both the models based on high or low volatility estimates. The statistical significance is substantial due to the fact of a huge number of tested scenarios. As mentioned earlier, the true model is expected to be significantly better in this situation for about 100 tested scenarios. The high volatility model outperforms the low volatility model, but the statistical significance of this result is small and it took a large number of simulations to show a small advantage of the high volatility model. However, for practical purposes, the two models do not substantially differ.

The final Table 2 illustrates the performance of the three different approaches presented in this paper—the matching based on the logarithmic utility function covered extensively earlier in this section, the matching based on the exponential utility function, and the Kullback-Leibler divergence—when applied to the comparison of the true versus high volatility model. We list the resulting estimate of E[V(T)], the estimate σ(V(T)), the information ratio $\frac{\mu }{\sigma }$ and the smallest number of observations *n* such that the comparison statistic ${\bar{V}}_{n}$ is positive with at least 95% probability. The matching algorithms based on the logarithmic utility function and the exponential utility function are nearly identical, but the logarithmic utility matching turned out to be slightly better both in terms of a higher expectation and a smaller standard deviation. This means that the true model can be identified faster for the logarithmic utility in comparison to the exponential utility. The Kullback-Leibler divergence gives values that correspond to adding the exposures without trading off the past discrepancies, which means that the longer lasting discrepancies are added repeatedly. As a result, both the expectation and the standard deviation are inflated in comparison to the utility matching algorithms. The absolute values are not important, what matters for statistical estimation is the resulting information ratio, which is about one third of the logarithmic utility matching information ratio. This means that it requires substantially more observations to correctly identify a better model. It would require 608 observations for the Kullback-Leibler divergence in comparison to the 90 observations for the logarithmic utility matching to assure that the event ${\bar{V}}_{n}>0$ has more than 95% probability.

## 5. Conclusions

This paper presents a novel method of measuring the quality of the probabilistic estimates that can be also applied in a time varying setting. This method is based on trading any discrepancy of the estimates on a hypothetical market, where both agents are maximizing their utility functions. The central result in this paper, Theorem 2, assures that the agent that uses true probabilities will realize a profit in expectation when trading against any other estimates. This result holds regardless of the choice of the utility function. The expected profit resulting from this procedure can be viewed as information gained in terms of statistical divergence.

## Figures and Tables

**Table 1:** Model Comparison, Different Volatilities.

Models	$\bar{V}$	s.d.	*t* Statistics	*p*-Value
True-High	0.00823793	0.00004757	173.16679	0.00000000
True-Low	0.01195865	0.00007445	160.61887	0.00000000
High-Low	0.00020800	0.00011238	1.850889	0.06418552

**Table 2:** True-High Model Comparison, Different Trading Approaches.

Divergence Model	$\bar{V}$	s.d.	Information Ratio	n: $P({\bar{V}}_{n}>0)=0.95$
Logarithmic	0.00823793	0.047572265	0.17314444	90
Exponential	0.00758190	0.048691108	0.15571435	112
Kullback-Leibler	0.81085788	12.15414317	0.06671452	608
